# Effectiveness of a Telecare Physical Therapy Program in Improving Functionality in Children and Adolescents with Cerebral Palsy: A Cases Study

**DOI:** 10.3390/children10040663

**Published:** 2023-03-31

**Authors:** Isabel Rodríguez-Costa, Vanesa Abuín-Porras, Paula Terán-García, Andrea Férez-Sopeña, Victoria Calvo-Fuente, Concepción Soto-Vidal, Soraya Pacheco-da-Costa

**Affiliations:** 1 Humanization in the Intervention of Physiotherapy for the Integral Attention to the People (HIPATIA), Physical Therapy Degree, Department of Nursing and Physical Therapy, Faculty of Medicine and Health Sciences, Universidad de Alcalá, Autovia A2, km 33.200, Alcalá de Henares, 28805 Madrid, Spain; isabel.rodriguezc@uah.es; 2 Neuromusculoskeletal Physical Therapy in Stages of Life Research Group (FINEMEV), Physical Therapy Degree, Department of Nursing and Physical Therapy, Faculty of Medicine and Health Sciences, Universidad de Alcalá, Autovia A2, km 33.200, Alcalá de Henares, 28805 Madrid, Spain; paulaateran@gmail.com (P.T.-G.); andrea.ferez@edu.uah.es (A.F.-S.); victoria.calvo@uah.es (V.C.-F.); conchi.soto@uah.es (C.S.-V.);; 3 Faculty of Sport Sciences, Universidad Europea de Madrid, 28670 Madrid, Spain; 4 Fundación DACER, Área de I+D+I, 28702 Madrid, Spain

**Keywords:** physiotherapy, cerebral palsy, action observation therapy, telerehabilitation, gross motor function, balance

## Abstract

Cerebral palsy (CP) is the most common physical disability in childhood and results in motor impairment that is often associated with other disorders. The aim of this study was to assess whether a telecare intervention consisting of Action Observation Therapy with a family-center approach produces improvements in functionality in children and adolescents with CP. Seven girls with CP ages between 6 and 17 participated in this case series study that lasted 12 weeks: 6 weeks of telecare program with a total of six sessions; and a follow-up period of 6 weeks. The outcome variables were Gross Motor Function (Spanish version of the Gross Motor Function Measure), balance (Spanish version of the Pediatric Balance Scale), walking endurance (6-min walk test) and walking speed (10-m walk test). The variables were measured before starting the study, after 6 weeks of intervention and after the 6-week follow-up period. Results showed statistically significant improvements in gross motor function (*p* = 0.02) after the intervention. After the follow-up period, gross motor function remained statistically significant (*p* = 0.02), as well as balance (*p* = 0.04) and walking endurance (*p* = 0.02). These results show that a telecare program has been beneficial in improving functionality with enhancements in gross motor function, balance and endurance in children and adolescents with CP that will facilitate participation.

## 1. Introduction

Cerebral Palsy (CP) is one of the most common causes of motor impairment in childhood [1], and it has an estimated overall prevalence of 2.0–3.0 cases per 1000 live newborns [2,3]. These motor alterations have a great impact on functional activity and participation [4].

CP mainly affects muscle strength and tone, altering motor and postural control [5]. Postural control can be defined as the ability to control position in space for stability and orientation purposes. It depends on the capabilities of the neuromuscular and musculoskeletal systems. The restricted ability to coordinate the muscles in postural synergies gives rise to dysfunctions in the activation of the postural response and in the adaptation of the posture in space, creating limitations in motor skills that require balance, such as walking or upper limb activities [6]. The gait alteration is conditioned by the area and location of the damage, affecting 90% of children with CP and producing a decrease in walking speed, endurance and cadence [7,8]. Consequently, motor and postural alterations cause limitations in functional activities related to daily life, additionally resulting in fear of falls and restrictions in participation [5].

In the pediatric field, telecare refers to the use of technology to provide remote support to children, families or people around them, promoting their health, functioning and development. Options for carrying out telehealth include computer-mediated communication, videoconferencing, phone calls, and text messages [9]. Because of the pandemic, the World Confederation for Physical Therapy (WCPT) promoted the use of telecare as a way to improve patient accessibility to Physical Therapy (PT) services [10,11]. This way of working is also a resource that can be applied in extreme situations such as the global pandemic caused by COVID-19, in geographically remote regions, or in patients with physical, financial and logistical difficulties [12,13]. The use of telecare allows PT interventions to take place within the natural environment of children and adolescents with CP and can be carried out based on functionality and from a real and significant approach [9], improving even motor function scores traditionally associated with in-person intervention [10]. In addition, this allows a family-centered approach in which services provided to children with disabilities should be based on the partnership between parents and professionals through equality, reciprocity and teamwork in order to provide care that is positive for both the child and his or her family [14] to promote children functionality.

One of the interventions which can be used in telecare is Action Observation Therapy (AOT). Through the activation of mirror neurons, AOT seeks the functional recovery of children and adolescents with CP through the restoration of damaged neural structures or the activation of complementary structures [14], and the observation of daily actions has shown good results in this population [14,15,16,17,18]. In addition, the development of telecare offers the opportunity to transfer AOT programs to the natural environment of children and adolescents through the use of videos or telecare sessions. The involvement of families in such programs can complement the PT intervention and improve function and satisfaction [19,20,21]. In order to make the telecare session more attractive, the videos must include actions of daily life adapted to the age and interests of children and adolescents with CP [14,20].

Therefore, children and adolescents with CP have their functionality reduced as a consequence of impairments of the neuromusculoskeletal system and gait function alteration. The aim of this study is to check whether a PT intervention by telecare consisting of a family-centered program with Action Observation Therapy in natural environments is effective in improving the functionality of children with CP. We hypothesized that the inclusion of a family-centered approach in an AOT intervention would add benefits to a telecare program for children with CP.

## 2. Materials and Methods

### 2.1. Study Design

The study was designed as a Case Series. This study presents the outcomes of the first group of the randomized clinical trial named Effectiveness of Physiotherapy for improving participation, gait and balance in children and adolescents with Cerebral Palsy, which is approved by the Animal Research and Experimentation Ethics Committee of the University of Alcalá (CEI/HU/2020/39) and it is registered in the database www.clinicaltrials.gov with number NCT04778930. The results of this preliminary study will also be included in the randomized clinical trial as a part of a larger group.

### 2.2. Participants

Seven girls diagnosed with CP aged between 6 and 17 years old.

Inclusion and exclusion criteria: children and adolescents with CP who are levels I-III of Gross Motor Function Classification (GMFCS) [22]; Manual Abilities Classification System (MACS) [23]; and Communication Function Classification System (CFCS) [24]; aged 6–17 years old. Children and adolescents will be excluded if they underwent surgery on lower limbs in the 6 months prior to their participation in the study, had surgery during the study, were unable to follow the planned program due to moving to another city, or other reasons.

The sample was recruited across Primary and Secondary Schools in the Community of Madrid and in the province of Guadalajara, Spain.

Six girls were in level II in the GMFCS, and 1 was in level I. Regarding age, there was a 7-year-old girl, and the other participants’ ages ranged from 12 to 17 years old. All of them had a diagnosis of spastic cerebral palsy. Their school modality was Integration, which means they attended classes with normally developing children of their age.

### 2.3. Variables

The variables in this study were collected at 3 points in time. An initial assessment (A1) was performed before starting the study, a final assessment (A2) at 6 weeks at the end of the intervention, and a follow-up assessment (A3) at 3 months.

Outcome variables:

- **Gross motor function**: measured with the Spanish version of the Gross Motor Function Measure (GMFM-SP) [25]. The Gross Motor Function Test (GMFT) is a commonly utilized standardized assessment tool to evaluate the gross motor abilities of children who have cerebral palsy. The Gross Motor Function Test (GMFT) is a commonly utilized standardized assessment tool to evaluate the gross motor abilities of children who have cerebral palsy. The minimum clinically relevant difference in those children and adolescents with levels II and III on the GMFCS is 1.5% and 1.2%, respectively [26]. Specifically, dimensions D and E were measured, as they were the goal dimensions for this study.
- **Balance**: measured with the Spanish version of the Pediatric Balance Scale (PBS). It is a modified version of the Berg Balance Scale and is used to measure balance in school-age children [27]. The PBS measures a child’s balance during specific functional tasks such as standing on one foot, standing on tiptoes, and reaching for objects while standing. The test is administered by a trained healthcare professional and typically takes around 20–30 min to complete.
- **Walking Endurance**: measured by the 6-min walk test to measure the maximum distance in meters that the individual can cover during this period of time [28]. The 6MWT involves measuring the distance a person can walk in 6 minutes, with the individual walking at their own pace. The test is typically administered by a trained healthcare professional and takes place in a quiet, flat area with a clearly marked walking path.
- **Walking speed**: measured by the 10-m walking test, which measures the time in seconds it takes the participant to walk 10 m at his or her own pace in a straight line [29].

### 2.4. Procedure

This case series study was carried out with an online intervention at the BlackBoard platform system of Alcalá University.

- **Initial assessment (A1)**: the collection of sociodemographic, anthropometric and outcome variables: gross motor function (Dimensions D and E of the GMFM-SP), balance (PBS) and gait (10 m walk and 6-min walk test).
- **Therapeutic Intervention**: 6 sessions of an online PT intervention, once a week, for 6 consecutive weeks.
- **Intermediate assessment (A2)**: data collection of outcome variables after 6 weeks of intervention.
- **Follow-up assessment (A3):** data collection of outcome variables after 12 weeks of the beginning of the intervention.

All assessments were performed at the Physical Therapy Unit at the University of Alcala, in the same room, at the same time of the day (after school time), and by the same Physiotherapist evaluator. Besides, in order to minimize possible bias, families were advised to bring their children and adolescents with the same kind of clothes (sportswear) and footwear during the pre-post intervention and the follow-up assessments. The study took place during the last trimester of 2022, with minimum variations in the climate conditions that could have interfered with the results

### 2.5. Telecare Educational Program

Session 1: A group session was conducted with participants and their families to explain and understand the concept of CP. The session encouraged the participation of children, adolescents, and their families/caregivers with a PowerPoint presentation to establish a common framework. The concept of CP and its causes were discussed in a simple and interactive way. Subsequently, there was a general sharing of specific objectives to generate a group feeling, and the doubts of participants and their families were resolved. Finally, the contents of the session were evaluated through a Kahoot!

Session 2: The main objective was to empower families to manage their children with CP. This individual session conducted an initial interview with participants and their families to explore and assess their physical, family and social environment and hobbies. The family-centered model emphasized the importance of involving families in the development of goals and tasks in the Physical Therapy intervention to promote collaboration with the treatment team’s guidelines. The session provided opportunities to discover what participants want to achieve, clarify what they need to achieve those goals, recognize what they already know and what they can do, and define what they still need to learn to improve their functional activity and participation in their environment. A Google form was sent to families by email to evaluate the session.

Sessions 3, 4, and 5: Group sessions were conducted to learn about AOT and its benefits, such as observation of actions performed by other individuals to facilitate activation of the same neural structures involved in the execution of the observed actions. Videos were used with actions familiar to participants, according to age and functional level, related to aspects of walking in their natural environment. Session 3: Actions observed in the video took place inside a family home, such as getting out of bed, walking to the dining room, picking up a story/book, sitting down to read, getting up from the armchair, walking to the kitchen, making a sandwich, returning to the dining room, and sitting on the armchair. Session 4: Actions were observed in an open-controlled environment to increase motor difficulty, such as leaving the house, walking through the housing community, going down a ramp, entering the housing community, going up a ramp, and walking to the housing portal. Session 5: Actions were observed in an open-random environment to increase the difficulty of the task, such as crossing a crosswalk, walking to the bus stop, waiting for the bus, going outside, walking to the park, and sitting on a bench/swing. Families were asked to record and send a video of participants performing one of the actions observed in the previous sessions to evaluate sessions 3, 4, and 5, which would be the basis for the final evaluation of the program.

Session 6: The final session discussed changes in functionality and participation in the environment following the previous sessions with each participant and their family. The doubts of participants and their families/caregivers were consulted and resolved, and a Google form was sent to families by email to evaluate the feasibility, acceptability, and the most important concepts of the educational program in the short term. An information leaflet was given to each participant with the most relevant information and guidelines of the intervention to continue performing and training the AOT activities proposed in the program in their natural environment.

### 2.6. Statistical Analysis

A descriptive analysis was performed: the numerical variables were presented with the mean and standard deviation, and the nominal variables with the median and interquartile range. The Wilcoxon test was used to carry out the pre-post analysis since it is a non-parametric test because the sample was small.

## 3. Results

Descriptive data are presented at baseline (Table 1) and for each of the assessments, both at the beginning of the study (A1) and at the end (A2), and the follow-up assessment after 3 months (A3).

Table 2 presents the results related to the objective dimension of gross motor function [(dimension E + dimension D)/2]. Under these data, each of these dimensions is shown individually, the score obtained in the Pediatric Balance Scale, the meters covered in the 6-min walk test and the seconds it took to perform the 10-m walk test both at the beginning (A1), at the end (A2) and three months after the end of the study (A3).

Table 3 shows the effect of the intervention in the short term. With regard to Gross Motor Function, it can be observed how changes have been produced in both dimensions (D and E); therefore, changes are also observed in the objective section corresponding to [(dimension E + dimension D)/2], all these changes have proved to be statistically significant with a *p* ≤ 0.05.

In relation to the Pediatric Balance Scale, improvements have been observed in the score of four of the participants, and these data have not been statistically significant (*p* = 0.11) but clinically significant. Something similar occurs with measurements in meters of the 6-min walk test; favorable changes were observed in five of the participants; however, these changes were not statistically significant (*p* = 0.20).

Finally, the results obtained in the 10-m walk test showed improvements in six of the participants in the time it took them to cover this distance, although these results were not statistically significant either (*p* = 0.09).

Table 4 reports the effect of the intervention in the long term. Regarding Gross Motor Function, statistically significant changes (*p* = 0.02) were obtained in the objective section. On the other hand, contrary to the results obtained in the short-term intervention, there were statistically significant changes in the Pediatric Balance Scale and in the 6-min walk test, with a *p* of 0.04 and 0.02, respectively. Finally, the 10-m walk test showed clinically significant results in five of the participants but were not statistically significant (*p* = 0.12).

## 4. Discussion

In this study, a total of 6 sessions of AOT combined with a family-center approach have been carried out, achieving statistically significant improvements in gross motor function and clinical changes in balance, gait speed and endurance in the short term. In addition, in the long term, balance and endurance have also shown significant improvements.

In the recent bibliography about telecare programs in the young population, there is some variability in terms of the duration of the programs and the frequency and duration of the sessions in the different studies. While the study by Cristinziano et al. [30] was carried out during the COVID-19 pandemic in 50-min sessions under the supervision of a therapist, without [31] was carried out in five sessions per week of 30 min each for 8 consecutive weeks. On the other hand, both Molinaro et al. [18] and Beani et al. [20] agreed with the implementation of a three-week intervention, carried out in five sessions per week. However, they differ in the duration of the sessions [18,20]; while Molinario et al. [18] carried out 90-min sessions, Beani et al. [20] carried out 45–60 min sessions. Therefore, there is no consensus in the literature on the optimal duration of the interventions, but they are similar to the one proposed in this research.

Due to the small amount of evidence found on telecare programs in CP, other studies have been consulted in which telecare interventions are carried out in persons with other pathologies. There is still great variability in terms of the duration of the programs and frequency of the sessions. Gagnon et al. [32] carried out an intervention in young people with arthrogryposis multiplex congenita by means of a home-based exercise program of 12 weeks duration, with three sessions per week of 15–30 min, as did Kenis–Coskun et al. [33], who developed a physical activity program for children with cystic fibrosis, performing three sessions per week for 12 weeks. On the other hand, the study conducted by Holthe et al. [34] in children with acquired brain injury lasted 20 weeks, including a total of 12 sessions, which included family sessions, school meetings, and group seminars.

Regarding the application of AOT, a systematic review conducted by Abdelhaleem et al. [35] stated that the duration of AOT programs usually ranges from 2 weeks to 12 weeks, with three to five sessions per week of one session per week, with sessions lasting between 15 and 60 min. Concerning video viewing and the execution of each action, in a study by Molinaro et al. [18], each video was viewed for 9–12 min, and each action was carried out for 6–8 min. In research by Buccino et al. [16,18], each video was viewed for 9–12 min, but the action was carried out for 2 min. However, in most of the literature consulted [15,20], both the viewing of the videos and the execution of the action took 2 min, whereas the visualization of the videos and the execution of the actions were performed for 3 min each. In addition, the videos used in the studies have selected actions that were attractive and adapted to the age of the participants, divided into simple motor tasks ordered according to their complexity and visualized in different perspectives: 1st person, 3rd person and from a lateral plane [15,16,18,20]. In the present study, the duration of the videos was 3 min, and the execution of each action was carried out from the lateral plane [15,16,18,20,36]. Each action was carried out three times without interruption, with each participant being asked to repeat the actions visualized in the videos 3–5 times between sessions. At the end of the sessions, each participant recorded and sent a video of him/herself performing one of the actions proposed during the program. Intervention videos were made on subjects of similar age to that of the participants. The actions were selected according to the needs and preferences of the participants and according to their age range and were ordered according to the complexity of the motor task.

Regarding the sessions with AOT, the present study has conducted an AOT with a family-centered approach. The videos used lasted approximately 3 min and presented actions that were familiar to the participant and related to the children’s natural environment, with the aim of encouraging participation.

In the literature consulted about AOT in Cerebral Palsy, Buccino [16] conducted a 3-week study, applying one session per day for 5 days a week, as did the research of Sgandurra [37], who also conducted an intervention of 15 consecutive sessions, performing five sessions per week. On the other hand, the study by Simon-Martinez [36] lasted 9 days with a total of 54 h of therapy. In another study conducted by Kirkpatrick [21], the AOT Therapy was not carried out by watching a video, but parents acted as models for their children and performed the different motor acts. Finally, some of these studies combined OAT with other therapies, such as Constraint Induced Movement Therapy using a splint on the less affected upper limb [36] and Mirror Therapy [38].

This intervention, in addition to carrying out AO, has developed an approach centered on the family. Currently, the family-centered model directed to specific tasks and meaningful goals has proven to be beneficial both in children with different neurodevelopmental diseases and in their families [39,40,41]. It has been proven that this approach is effective in improving the development of functional skills, psychological state, gross motor function and static and dynamic balance [39,42], as well as increasing satisfaction and participation in physical activities in children and adolescents with functional diversity [41]. Moreover, this framework allows the empowerment of families and the enhancement of the knowledge and management of the health condition of their children with functional diversity [41]. Therefore, the improvement of their psychological state [39,40]. In general, increasing the satisfaction of the families of children with CP with regard to their participation in the home and community [43].

Regarding the results of the intervention, this study has shown statistically significant differences in three of the four variables that were measured both in the short and long term. Balance and endurance showed improvement in the long term, whereas the increase in gait speed was not maintained over time. This can be related to the loss of muscle strength that generally accompanies a decrease in physical activity [44]. Gross motor function was measured using the Spanish version of the Gross Motor Function Measure since it is the main scale for assessing gross motor function in children with Cerebral Palsy and has demonstrated high levels of validity and reliability in clinical and research settings [45] In this variable the results have shown statistically significant changes in the objective dimension, and in the D and E dimensions in the short term, while in the long term, these changes have been maintained in the objective dimension, while the D and E dimensions have shown improvements but clinically significant. The study by Jeong [46] also carried out a program using AOT of six weeks duration. Its results showed significant differences in gross motor function measured with the GMFM-88. Moreover, the study conducted by Jung [47] performed a four-week intervention combining AOT with vibration in children with spastic Cerebral Palsy, revealing statistically significant improvements in GMFM scores in D and E dimensions.

The Spanish version of the Pediatric Balance Scale (PBS) was used to measure balance. This variable showed statistically significant long-term changes. The study by Jung [47] also measured balance with PBS and, after 4 weeks of intervention, showed significant changes. On the other hand, Jeong [46] also measured balance using the Pediatric Reaching Test (PRT) and, after 6 weeks of intervention with TAO, showed significant changes in dynamic balance. Other studies have also assessed balance in different neurological pathologies; Pelosin [46] assessed balance in patients with Parkinson’s disease using the Berg scale after 5 weeks of therapy with action observation showing positive changes in the results after the intervention. Likewise, the study by Moon [47] also showed changes in balance variables in chronic stroke patients after 4 weeks of action-observation training.

Regarding walking, the time in seconds was measured in the 10-m walk test, and the distance covered in meters in the 6-min walk test. The results showed significant improvements in the 6-min walk test in the long-term measurement, while the 10-m walk test only obtained clinically significant improvements; likewise, Jung [46] measured both variables after a TOA program in children with cerebral palsy and evidenced significant improvements in the results of both tests and in the results of the Time Up and Go test.

The results of the current study are consistent with previously conducted studies that confirm that an intervention using AOT is effective for the improvement of gross motor function, balance and walking performance in Cerebral Palsy. The content of the program was quite novel, as it combined AOT activities [36,37,38] that have already shown benefits in the motor area, especially in upper limb function [36,38], adapted to the age and specific conditions of the participants and objectives of the study, with family-centered practices [39,40,41,42,43]. Each part of the intervention was focused on the improvement of the variables of the study (balance, walking endurance, walking speed and dimensions related to the walking ability of the GMFT), with positive results. To the authors’ knowledge, this is the first study this study confirms the importance of an action-observation telecare intervention with a family-centered approach in line with the global tendencies in current lines of practice that enhance the importance of involving the family in every clinical intervention, even those that seem highly dependent of the individual, such as AOT. Clinicians could consider adding up this kind of program to their in-person interventions or using them as main interventions in specific cases when the displacement of the children is not possible due to distance or scheduling difficulties.

### 4.1. Limitations

The main limitation encountered during this study was the lack of adherence to the treatment by some participants. On the other hand, the younger participants had greater difficulty in maintaining attention and participating actively during the session, in comparison with the adolescents, who, in addition to being more motivated in carrying out the activities, had greater interaction among themselves throughout the weeks, creating affective bonds with the other participants in their group. Moreover, the difficulty to coincide in schedules with the different families and their misunderstanding of the use of electronic devices and the video-call tool used for the sessions was a limitation that interfered with the development of the different activities. Finally, it has to be taken into account that case-series studies have the disadvantage of section bias and difficult comparability, and cases may not be generalizable due to the lack of controls

### 4.2. Future Research Lines

In future lines of research, it would be interesting to develop new studies that combine Action Observation Therapy with different therapies in order to prove its efficacy in the treatment of Cerebral Palsy. On the other hand, regarding telecare programs, it is important to look for effective options to increase adherence to treatment, especially in younger subjects, who have shown more difficulties in staying attentive and focused during the sessions.

## 5. Conclusions

This study has shown that a telecare program using AOT with a family-centered approach is effective in improving functionality with improvements in gross motor function, balance and gait in children with Cerebral Palsy.

Likewise, the results show that telecare is an effective tool to complement rehabilitation in a virtual way. More studies with a larger sample of participants are needed to corroborate the efficacy of the therapy in children and adolescents with cerebral palsy.

## Figures and Tables

**Table 1:** Sample Baseline Description.

Variable	*n*
Sex	
Women	7
Men	0
Gross Motor Function Classification System	
Level II	6
Level III	1
Age	
6–11 years	1
12–17 years	6
Education/schooling	
Integration (number of subjects)	7
Special education (number of subjects)	0

**Table 2:** Description of outcome variables.

	Participants								
	1	2	3	4	5	6	7	Mean	Standard Deviation
Variables A1	A_1_								
Gross Motor Function *	48.66	96.63	78.63	77.51	65.37	70.50	92.09	75.62	16.24
Dimension D	48.71	97.43	79.48	89.74	66.66	74.35	89.74	78.01	16.60
Dimension E	48.61	95.83	77.77	65.27	63.89	66.36	94.44	73.21	17.22
Balance **	41	55	46	42	48	48	54	47.71	5.37
Walking endurance (meters) ***	140	666	400	372	250	330	336	356.28	161.89
Walking Speed (meters/second) ****	21.18	4.14	4	9.56	15.4	7.18	4.38	9.40	6.60
Variables A2	A_2_								
Gross Motor Function *	55.28	98.02	86.16	78.20	65.37	71.79	94.57	78.48	15.57
Dimension D	56.41	97.43	82.05	89.74	66.66	76.92	92.31	80.21	14.70
Dimension E	54.17	98.61	90.27	66.66	63.89	66.66	96.83	76.72	17.99
Balance **	46	54	55	46	48	49	53	50.28	3.94
Walking endurance (meters) ***	145	642	568	380	250	335	360	368.57	158.12
Walking Speed (meters/ second) ****	19.30	3.67	3.54	7.38	13.45	7.9	4.10	8.47	5.91
Variables A3	A_3_								
Gross Motor Function *	56.67	98.02	80.71	83.06	65.37	71.79	98.02	79.09	15.70
Dimension D	56.41	97.43	79.48	89.74	66.66	76.92	97.43	80.58	15.51
Dimension E	56.94	98.61	81.94	76.38	63.89	66.66	98.61	77.57	16.52
Balance **	46	54	50	47	48	49	54	49.71	3.19
Walking endurance (meters) ***	141.6	702	414	380	268	335	370	372.94	171.26
Walking Speed (meters/second) ****	21.22	2.98	4.13	9.5	13.02	7.1	4.00	8.85	6.50

* Spanish version Gross Motor Function Measure (%); ** Spanish version Pediatric Balance Scale (points); *** 6 min walk test (meters); **** 10 m walk test (meters/seconds).

**Table 3:** Short-term effect of the intervention V_2_ − V_1_.

Variables	P1	P2	P3	P4	P5	P6	P7	*p*+
Gross Motor Function *	6.62%	1.39%	7.53%	0.69%	0%	1.29%	2.48%	0.02
Dimension D	7.7%	0%	2.57%	0%	0%	2.57%	2.57%	0.05
Dimension E	5.56%	2.78%	12.5%	1.33%	0%	0%	1.39%	0.04
Balance **	5	−1	9	4	0	1	0	0.11
Walking endurance (meters) ***	5	−24	68	8	0	1	5	0.20
Walking Speed (meters/second) ****	−1.88	−0.47	−0.46	−2.18	−1.95	0.72	−0.28	0.09

* Spanish version Gross Motor Function Measure (%); ** Spanish version Pediatric Balance Scale (points); *** 6 min walk test (meters); **** 10 m walk test (meters/seconds); + Wilcoxon Test.

**Table 4:** Long-term effect of the intervention V_3_ − V_1_.

Variables	P1	P2	P3	P4	P5	P6	P7	* p+ *
Gross Motor Function *	8.01%	1.39%	2.08%	5.55%	0%	1.29%	5.93%	0.02
Dimension D	7.7%	0%	0%	0%	0%	2.58%	7.69%	0.11
Dimension E	8.33%	2.78%	4.17%	11.11%	0%	0%	4.17%	0.06
Balance **	5	−1	4	5	0	1	0	0.04
Walking endurance ***	1.60	36	14	8	18	5	34	0.02
Walking Speed ****	0.04	−1.16	0.13	−0.06	−2.38	−0.08	−0.38	0.12

* Spanish version Gross Motor Function Measure (%); ** Spanish version Pediatric Balance Scale (points); *** 6 min walk test (meters); **** 10 m walk test (meters/seconds); + Wilcoxon Test.

## Data Availability

Data available under reasonable request.
